# Cancer Management during COVID-19 Pandemic: Is Immune Checkpoint Inhibitors-Based Immunotherapy Harmful or Beneficial?

**DOI:** 10.3390/cancers12082237

**Published:** 2020-08-10

**Authors:** Silvia Vivarelli, Luca Falzone, Caterina Maria Grillo, Giuseppa Scandurra, Francesco Torino, Massimo Libra

**Affiliations:** 1 Laboratory of Translational Oncology, Department of Biomedical and Biotechnological Sciences, University of Catania, 95123 Catania, Italy; silvia.vivarelli7@gmail.com; 2 Epidemiology Unit, IRCCS Istituto Nazionale Tumori ‘Fondazione G. Pascale’, I-80131 Naples, Italy; 3 Otolaryngology Unit, Department of Medical Sciences, Surgical and Advanced Technologies, University of Catania, 95123 Catania, Italy; grillo.caterinamaria@gmail.com; 4 Medical Oncology Unit, Azienda Ospedaliera Cannizzaro, 95126 Catania, Italy; giusy.scandurra@gmail.com; 5 Department of Systems Medicine, Medical Oncology, University of Rome Tor Vergata, 00133 Rome, Italy; torino@med.uniroma2.it; 6 Research Center for Prevention, Diagnosis and Treatment of Cancer, University of Catania, 95123 Catania, Italy

**Keywords:** COVID-19, SARS-CoV-2, cancer, immune-checkpoint inhibitors, Anti-PD-1 monoclonal antibody, immunotherapy

## Abstract

The coronavirus disease 2019 (COVID-19) is currently representing a global health threat especially for fragile individuals, such as cancer patients. It was demonstrated that cancer patients have an increased risk of developing a worse symptomatology upon severe acute respiratory syndrome associated coronavirus-2 (SARS-CoV-2) infection, often leading to hospitalization and intensive care. The consequences of this pandemic for oncology are really heavy, as the entire healthcare system got reorganized. Both oncologists and cancer patients are experiencing rescheduling of treatments and disruptions of appointments with a concurrent surge of fear and stress. In this review all the up-to-date findings, concerning the association between COVID-19 and cancer, are reported. A remaining very debated question regards the use of an innovative class of anti-cancer molecules, the immune checkpoint inhibitors (ICIs), given their modulating effects on the immune system. For that reason, administration of ICIs to cancer patients represents a question mark during this pandemic, as its correlation with COVID-19-associated risks is still under investigation. Based on the mechanisms of action of ICIs and the current evidence, we suggest that ICIs not only can be safely administered to cancer patients, but they might even be beneficial in COVID-19-positive cancer patients, by exerting an immune-stimulating action.

## 1. Introduction

Since December 2019, a severe pneumonia outbreak diffused from a fish market in Wuhan (Hubei province, China) to all over the World, being declared a pandemic on the 11 March 2020 by the World Health Organization (WHO, Geneva, Switzerland) [1].

The pandemic is caused by the severe acute respiratory syndrome associated coronavirus-2 (SARS-CoV-2), identified as the causal agent of the so-called coronavirus disease 2019 (COVID-19) [2]. Presumably originated in bats and then diffused and adapted to infect the human host, the SARS-CoV-2 belongs to the *Coronaviridae* family and to β-coronavirus genus. It is the third known CoV, following the severe acute respiratory syndrome coronavirus (SARS-CoV) and Middle East respiratory syndrome coronavirus (MERS-CoV) to represent a threat for humans, and it is the first one to cause a pandemic in this 21st Century [3].

Although the biomedical research is currently proceeding at an exceptionally fast-pace, no vaccine and no effective therapies are currently available, but only treatments based on the COVID-19 symptomatology [4,5].

COVID-19 symptoms may include the viral infection of the lower respiratory tract, dry cough, fever and fatigue. The infection might be followed by pneumonia, associated either with mild complications, including dyspnea and lymphocytopenia, or with more severe outcomes, including a significant hypoxia, associated with the rise of the acute respiratory distress syndrome (ARDS) [6]. Because of the ARDS, a non-negligible proportion of COVID-19-affected patients might need to undergo oxygen therapy and to be admitted to an intensive care unit (ICU), as the ARDS might be followed by septic shock, multiorgan dysfunctions or death (in a 1% to 4% of the cases) [7].

The host’s infection is initiated by the SARS-CoV-2 spike (S) protein binding to the extracellular enzymatic domain of the transmembrane angiotensin-converting enzyme 2 (ACE2) cellular receptor, expressed by the epithelial and endothelial cells within the alveoli [8]. ACE2 is also expressed distally, in kidney, heart, intestine and neuronal cells, although it is not clear whether SARS-CoV-2 may directly infect these distant targets [9].

The COVID-19 symptomatology is very heterogeneous and strictly depends on the nature of the interaction occurring between SARS-CoV-2 and the host. SARS-CoV-2 depending-factors are: Specific genetics, titer and virulence. Host-depending factors are: Genetics (in particular the HLA genes), age, gender, humoral and cellular immune system health and, finally, the concurrent presence of comorbidities [10]. Subsequently, the overall host response to SARS-CoV-2 invasion might be classified in four types: (1) mild: With upper respiratory tract infection and digestive tract symptoms; (2) moderate: With lower respiratory tract infection, pneumonia, mild hypoxemia and presence of pulmonary lesions; (3) severe: With pneumonia and severe hypoxemia; and (4) critical: With ARDS, septic shock, myocardial injury, kidney injury and diffused coagulation issues [11].

In particular, following SARS-CoV-2 infection, there is a high rate of immune inflammatory cells that start to infiltrate the alveolar space and release cytokines (e.g., IFN-α, IFN-β, IFN-γ, IL-1β, IL-6, IL-8, TNF-α, TGFβ and GM-CSF) and chemokines (e.g., CCL2, CCL3, CCL5, CXCL8 and CXCL9), thereby resulting in an acute lung injury. If unresolved, this exacerbated production may lead to the so-called “cytokine storm” (CS) [12]. Among the most relevant cytokines in the CS manifestation, a key role is played by: IL-6, IL-1β and GM-CSF [13,14,15]. Several are the immune-modulating drugs currently tested off-label to contrast the CS in COVID-19 patients [16,17]. Locally, the CS may determine immune cells infiltration, the increase of inflammatory cell death in the alveoli, with a concurrent dysregulated permeability due to the extended tissue damage [18]. Additionally, the CS may induce the development of ARDS, respiratory failure and multiple organ failure, finally leading to death [19].

Following the first humoral immune response, the adaptive immune cellular response gets also triggered, with the B-cell activation and the production of IgM and IgG [20]. Moreover, T-cells are activated. Interestingly, in severe and critical COVID-19 cases, T-cells become dysregulated, hyperactivated and, finally, exhausted, thereby expressing on their surface markers of both activation (including CD69, CD38 and CD44), and exhaustion (such as mucin-3, PD-1 and NKG2A) [21]. Exhausted T-cells are few in number in COVID-19 patients, hence suffering from lymphocytopenia and being unable to eradicate the infection. As a consequence, the SARS-CoV-2 might spread within all the body, with the concurrent manifestation of viremia, septicemia and septic shock. This critical situation might be followed by myocardial and kidney injury, as well as coagulation dysfunction, which might lead to death [21].

A vaccine is under investigation and 13 clinical trials are currently opened at clinicaltrials.gov, with the common aim to test the efficacy of several different vaccinal strategies [22]. Analogously, an effective anti-SARS-CoV-2 therapy does not exist and a very high number of clinical trials are presently evaluating the efficacy of combining standard of care procedures with molecules that might show either a direct anti-viral effect or the ability to reactivate the immune system of the host against the virus. This immune system remodulation might either reduce the CS (e.g., anti-inflammatory molecules, antibodies blockers of pro-inflammatory cytokines) or reactivate the cellular-mediated defective immune response (e.g., immune checkpoint inhibitors (ICIs)) [23,24,25,26].

The consequences of COVID-19 may be really heavy for people with an immunocompromised immune response, including cancer patients. In this review, the issues of treating cancer during this difficult time of pandemic will be reported. The overall impact of COVID-19 on cancer management will be also analyzed, with reference to all the up-to-date published findings. One really yet debated question is the use of ICIs in cancer care during the pandemic, as they represent molecules which directly target and modulate the immune response. The present knowledge regarding this topic will be deeply dissected. Moreover, our personal perspective, sustained by the available clinical findings, will be also disclosed.

## 2. Cancer Patients and COVID-19: What Are the Global Consequences for Oncology?

The outcome of COVID-19 has been reported to be worse in patients with coexisting pathologies which are correlated with an impaired immune response to pathogens [7]. For example, elderly subjects or individuals with coexisting comorbidities including hypertension, obesity, diabetes or cancer, have a defective immune system that cannot efficiently contrast the SARS-CoV-2 infection. In those cases, COVID-19 might quickly degenerate towards a severe or critical status [7].

In particular, cancer patients are highly sensitive to infections in general, and they may be therefore vulnerable to SARS-CoV-2 too. Cancer itself may be linked with an extensive immunosuppressive status [27]. Additionally, the immunosuppressive condition might be exacerbated as a consequence of strong myelosuppressive therapies, such as chemotherapy or radiotherapy [28]. Given their immune-compromised status, cancer patients infected by SARS-CoV-2 might be at a higher risk of developing severe and critical consequences upon COVID-19, including ARDS, septic shock and acute myocardial infarction [29,30,31].

Cancer care at the time of COVID-19 has been defined as a war on two fronts, mainly due to the reorganization of resources against the COVID-19 emergency. Indeed, the data regarding cancer patients are still very few and incomplete. Consequently, oncologists are faced with a dilemma about how to balance the risk of COVID-19 exposure with delivering an effective therapy and assistance to patients [32]. On the other end, cancer patients are directly affected by COVID-19 healthcare system disruptions, as they may suffer from an increased perception of their fragility. This is mainly caused by the distance from care centers, with telemedicine substituting the in-presence visits. Additionally, problems of cancellation and rescheduling might arise, plus difficulties coming with the imposed self-isolation during the lockdown [32].

Mauri et al., on behalf of the International Oncology Panel and European Cancer Patient Coalition collaborators, on the 25 March 2020, published a summary of all the main international recommendations for patients with cancer during the COVID-19 pandemic, translated in 23 diverse languages [33]. The international scientific panel revised 63 different oncology guidelines from all over the world, thereby producing a univocal document, accessible from many different countries, as well as not English-speaking people. The panel of medical experts listed six main areas of recommendation, including: The relationship with the physician, the indications of hygiene and protective measures anti-COVID-19, the features of SARS-CoV-2 infection and the restrictions operated by cancer centers to prevent the spread of the virus [33].

Alongside, the oncologists need to reorganize their activity to respond to the threat and to protect their sensitive patients. Patients with cancer, including hematological cancers and those who received hematopoietic stem cell transplantation, are generally more vulnerable to pathogens because their immune system is impaired. In the case of COVID-19, as a consequence of the immunosuppression, they have a higher risk to develop severe and critical complications following SARS-CoV-2 infection [34]. Often the oncologists work in stressful conditions, given the reorganization of infrastructures and instrumentation for the management COVID-19 patients who need intensive care. The advice for oncologists is to adapt to these difficult challenges, hoping that the long-term consequences will be not too devastating for fragile individuals [34].

To help the oncologists, the main medical oncology societies, including American Society of Clinical Oncology (ASCO), European Society for Medical Oncology (ESMO) and Italian Association of Medical Oncology) AIOM, published several guidelines addressed to both oncologists and cancer patients, in order to safely guarantee the continuation of treatments and to ensure high standards of care. Overall, all the societies agreed on the design of specific therapeutic protocols to direct the route of accession to the hospitals for cancer patients. When possible, they are advised to suspend all the unnecessary treatments and to use the telemedicine for the follow-up visits or other evaluations [35,36].

In the same direction, the “Cancer Core Europe” (CCE), which includes seven European cancer institutes, such as the Italian Istituto Nazionale dei Tumori di Milano, recently published a perspective in Nature. CCE conceived a general policy to ensure the best standard of care for cancer patients during the pandemic, even with shortages in personnel, medications, beds and infrastructures [37].

Two main goals are evidenced in the guidelines: (1) to minimize hospital visits and hospitalization and (2) to prevent anti-cancer treatment complications. In details, to reduce the time spent by oncological patients in the hospital, it has been suggested: (1) to convert, where possible, to intravenous treatments to subcutaneous or oral administration, that can be pursued at home and (2) to postpone cancer non-emergency surgery or replace it with radiotherapy. Moreover, in order to prevent insidious therapy-linked side effects, such as neutropenia and lymphopenia, it was advised to reduce cytotoxic chemotherapy or to use palliative care instead of proper treatment where possible [37]. As it will be largely discussed later, it is unlikely that anti-cancer treatments, per se, increase COVID-19 infection risk. Nevertheless, cancer patients, when infected by SARS-CoV-2 might develop more severe outcomes, if anti-cancer treatments induce a weakening of the host immune health [38].

To help clinicians, consortia and registries play a pivotal role in collecting and ease the analyses of data coming from cancer patients infected with SARS-CoV-2. The US COVID-19 and Cancer Consortium (CCC19) is a multicenter registry which is currently helping to fill the gap in cancer care during this pandemic. In particular, CCC19 is assessing the number of cancer patients affected by COVID-19. At the moment CCC19 comprehends 90 institutions across 28 different US states plus Canada and Spain [39]. Furthermore, the UK Coronavirus Cancer Monitoring Project (UKCCMP), is a monitoring project aimed to centralize the data deriving from 55 different institutions within the whole UK. UKCCMP goal is the collection and analysis of clinical information from cancer patients affected by COVID-19 [40].

In cancer patients, multiple factors may contemporarily contribute to the observed increased prevalence and severity of COVID-19. Given the often-chronic nature of the disease, such individuals may be older or smokers. Furthermore, they might have comorbidities (preexisting, related to cancer and/or medications). Altogether, these risk factors may collectively contribute to the individual fragility of people who develop a tumor. In addition, myelosuppression and lymph-suppression in these patients might be triggered by the usage of cytotoxic drugs [41].

Given this very intricated clinical situation, the oncologist must evaluate case-by-case what to do to preserve cancer patients from COVID-19, as well as to guarantee a successful anti-cancer treatment. Sometimes delaying the treatment can be helpful, while other times the postponing may be even more deleterious [41].

## 3. Cancer Care and COVID-19: Clinical Observations

Since the beginning of the pandemic, already 70 clinical studies carried on cancer patients and related to COVID-19 have been registered at clinicaltrials.gov. These trials include both observational and interventional studies, aimed to shed more light on the association occurring between cancer and COVID-19. The search criteria employed were: (1) condition or disease: “Cancer” and (2) other terms: “COVID-19” (Search date: 30 June 2020). All the registered studies are reported in Table 1.

Despite the very short time passed since the beginning of this noxious pandemic, some clinical observations have been already published, although more robust data on bigger cohorts are further needed to confirm the preliminary assessments. In an earlier study published by Liang et al., in Lancet oncology, on the 14 February 2020, an analysis of the feature of SARS-CoV-2 infection in 18 cancer patients was conducted [42]. The study was performed in a very small group of patients in China, during the first appearance of the COVID-19. The limit of this study is, of course, the reduced number of cases analyzed. Overall, the authors observed an increase of the death outcome in infected cancer patients compared with the general SARS-CoV-2 infected population. Moreover, infected cancer patients were at higher risk of severe complications from COVID-19, if compared to patients without cancer. The authors concluded that, where possible, the chemotherapy or surgery may be postponed. Alternatively, where the delay is not possible, a stronger personal protection and other protective measures have been suggested [42]. Although the study conducted by Liang et al. was the first one to examine the correlation existing between cancer and COVID-19, it was afterward criticized because the cases analyzed were very few (only 18) and highly heterogeneous, therefore the results have been considered overall not conclusive [43].

Subsequently, in a larger study published by Yu and colleagues, in Jama Oncology, on the 25 March 2020, the transmission of SARS-CoV-2 in cancer patients was examined. Wherein, 1524 Chinese patients with cancer were analyzed and the incidence and outcome of COVID-19 infection were evaluated. Only 12 out of the 1525 patients contracted SARS-CoV-2 and developed COVID-19 (0.79%). Although a smaller number (of only 12 cases) have been analyzed, the overall incidence of COVID-19 in patients with cancer was found to be higher than in patients without cancer (0.32%, at the time of the study) [44]. Moreover, in patients with non-small cell lung cancer (NSCLC) over 60 years of age, the incidence of contracting SARS-CoV-2 infection was higher than in younger ones. No significant variations were observed among cancer patients in relation to their individual anti-cancer regime. The authors suggested, during the pandemic, to reduce the frequency of hospital visits in order to avoid a place where the likelihood to be infected is higher. Moreover, they encouraged to self-isolation and lockdown, where possible [44].

A retrospective study on Chinese cancer patients was performed, to characterize the clinical features of COVID-19-severely infected cancer patients. The study was published in Jama oncology by Zhang and colleagues, on the 26 March 2020 [45]. It is a very small analysis, conducted on 28 cancer patients with COVID-19. Tumor-affected patients presented poorer outcomes and a higher mortality rate compared to non-cancer patients. Moreover, the anti-cancer treatments, if received within 14 days prior the infection, increased the risk of developing severe symptoms, independently of the type of treatment. Given the limited number of subjects implicated in the analyses, the authors suggest the validation of these observations to larger cohorts of cases [45].

In contrast with the reported Chinese studies, a New York-based US study, published by Miyashita et al., in Annals of Oncology, on the past 21st of April, reported that there was no significant difference between the incidence and overall mortality rate of COVID-19 between cancer and non-cancer patients [46]. The authors analyzed a total of 5688 patients with COVID-19, among which 334 had cancer. The authors postulated that the impairment of the immune system in such patients might contrast the possible development of a CS during COVID-19 progression, thus explaining the surprising better outcome [46].

Contrariwise, in another study, cancer patients were assessed overall more vulnerable to SARS-CoV-2 infection, in agreement with the previously reported Chinese studies. The study, published in Cancer Discovery the 28 April 2020, by Dai et al., analyzed a cohort of 105 cancer patients and 536 normal patients, all affected by COVID-19. The 105 SARS-CoV-2 positive cancer patients (from the Hubei province) were all with active cancer at the period of study, between January 1st and February 24th. The analysis demonstrated that patients with cancer had higher risks of severe COVID-19 outcomes. In particular, patients with hematological cancer, lung cancer or with metastatic cancer (stage IV) had the highest severe events frequency and death rate [47]. The patients enrolled, received heterogeneous anti-cancer treatments and, in some cases, more than one type in combination. Interestingly, patients who underwent surgery or received ICIs, had a higher chance to develop severe symptoms therefore needing ICU hospitalization and controlled ventilation. The limitation of this latter observation is the very low number of analyzed cases (only six ICI-treated and four surgery-treated patients). Therefore, larger cohorts are needed to have a robust and meaningful indication about risks associated with specific anti-cancer interventions [47].

A clinical study performed by the Institute Curie (Paris, France), analyzed 141 cancer patients with COVID-19, amongst the 9842 cancer patients referred to the Institute between the beginning of January and early May 2020. This study claims to be undertaken not only on hospitalized subjects, like the others previously published. In line with the New York study conducted by Miyashita et al., the conclusions of this analysis are that COVID-19 incidence in cancer patients, when adjusted for age, sex and preexisting comorbidities, is not dissimilar to the one observed in the general population. The authors suggested that the outcome of COVID-19 is primarily driven by the initial severity of the infection, rather than by the presence of cancer condition [48].

Thanks to the previously described US and UK cancer registries, CCC19 and the UKCCMP, recent independent studies were published in the Lancet journal, on the 28th of May 2020, reporting observations made on bigger groups of cancer patients with COVID-19 (around 800 subjects) [49,50].

The first study, conducted by Lee and colleagues, analyzed data coming from the UKCCMP, a clinical registry including data coming from all the main cancer centers in the UK. All patients with active cancer were eligible for the study. The primary endpoint was death or dismission from the hospital. The study considered 800 patients, with active cancer and diagnosed positive to SARS-CoV-2, between March 18th and April 26th 2020. The 52% of them developed mild symptoms, the 28% died. Importantly, the risk of severe and critical COVID-19 manifestations was mainly associated with the older age [49].

In fact, as described, in cancer patients affected by COVID-19, the mortality is driven by a mix of factors such as age, male gender and presence of comorbidities. After adjusting for those variables, the authors found that chemotherapy received in the 4 weeks before COVID-19 diagnosis had no incidence on the overall rate of death. Furthermore, no significant effect on mortality was found for ICI-immunotherapy, hormone therapy, targeted therapy and radiotherapy received within the 4 weeks prior COVID-19 diagnosis [49]. Although the mortality of cancer patients was higher than the non-cancer population, such discrepancy was not due to the presence of active cancer nor to the received anti-cancer treatment. This difference was correlated to the other risk factors, including: Age, sex, smoking and coexisting comorbidities [49].

Kuderer and colleagues, analyzed data from USA, Canada and Spain cancer patients, deposited in the registry of CCC19. They collected data from baseline, since the detection of SARS-CoV-2 positivity and at 30-days following SARS-CoV-2 infection diagnosis. The assessment of mortality after the 30-day was the primary endpoint [50]. It is a study still ongoing (NCT04354701, see Table 1), and the results published are preliminary findings. As such, 928 cancer patients with COVID-19 were analyzed. The 30-day all-cause mortality was higher than in non-cancer COVID-19 affected population. The explanation given by the authors was that cancer patients may be immunocompromised by cancer and/or anti-cancer treatments. Older patients with comorbidities have a higher risk to develop severe COVID-19. By May 7, the 13% of the COVID-19 positive cancer patients enrolled died within the 30-days diagnosis SARS-CoV-2 positivity [50]. Additionally, the stronger association with 30-days all-cause death was present in patients receiving a combination of azithromycin plus hydroxychloroquine, drugs used to contrast the viral infection during the severe ARDS manifestations [50].

From these two studies some questions remain unsolved: (1) is any type of anti-cancer therapy a positive or a negative risk factor for SARS-CoV-2 infection? (2) are ICIs, in particular, a risk factor for SARS-CoV-2 infection rate and/or development of severe symptoms? In the UK study, no interaction with any of the anti-tumor therapies was found, and the higher death rate was instead associated with age, sex, smoking and comorbidities. Whereas, the US study is still open, and it did not analyze yet the correlation between the risk of infection/mortality and the anti-cancer regime used. In contrast with the earlier observations derived from the Chinese smaller studies, the two novel studies associated the increased risk of COVID-19 complications with age, sex and coexisting pathologies instead of cancer pathology itself. The main conclusion coming out from these two bigger studies published in the Lancet journal, is that oncological care should be offered to cancer patients during COVID-19 emergency, including chemotherapy [51].

The latest published clinical study is a US one, conducted on New York city cancer patients, hospitalized at the Memorial Sloan Kettering Cancer Center. A total of 423 cancer patients with COVID-19 over a total of 2035 were enrolled. In which, 40% of the positively tested were hospitalized, and 20% of them developed severe COVID-19, while 12% died. Factor predictors for ICU, oxygen need and severe outcomes were: Age and previous or ongoing treatment with ICIs. In contrast, chemotherapy and major surgery did not show any significant difference. Importantly, the endpoint considered was the hospitalization and admission to ICU, and not death rate. This association was found independently from age, sex and cancer type. This observation was confirmed in a heterogeneous subset of cancers, including lung cancer [52].

Since the beginning of this pandemic, nine independent clinical studies have been published about the risks possibly related to SARS-CoV-2 infection in patients with cancer. As reported, the risks analyzed often differed between the diverse studies, and included: (1) the risk of contracting the virus; (2) the probability of developing a severe symptomatology; and (3) the percentage of death [42,44,45,46,47,48,49,50,51,52].

These studies showed a number of limitations. First of all, the considered control group differs between studies. While some studies compared cancer patients with or without COVID-19, other ones considered COVID-19 patients with and without cancer. Additionally, the earlier results derived from very small cohorts, therefore being not very conclusive. Although more recent reports included a bigger number of cases, the conclusions reported were often not comparable between each other, as the endpoints examined were often not the same. For example, while Kuderer et al. contemplated the 30-days death toll as primary endpoint, the Sloan Kettering study investigated the incidence of ICU hospitalization [50,52].

Although the association between anti-cancer therapy and COVID-19 is still unclear, the observation reported consistently by the majority of the studies is that comorbidities, often co-existing in cancer patients, may predispose not necessarily to an increased incidence of the viral infection, but to develop more severe symptoms following the infection (such as ARDS). In general, since cancer patients have an overall heterogeneous clinical history, it is very difficult to discriminate between the selected effects that cancer and other pre-existing comorbidities might have on the individual immune response against SARS-CoV-2. In order to gain more robust data and fill this gap, further bigger (and more controlled) studies are ongoing, with the goal to shed light on the link existing between COVID-19 and cancer (Table 1).

## 4. COVID-19 and Anti-Cancer Therapy: Advantages and Disadvantages of ICI-Immunotherapy

The latest bigger studies deriving from CCC19 and UKCCMP data-registries evidenced that none of the anti-cancer therapeutic regimens may affect neither the rate of severe COVID-19 nor the mortality rate in cancer patients [51]. While a latest study conducted at the Memorial Sloan Kettering Cancer Center, highlighted, specifically for ICIs, an association with increased ICU admission rate, but not death rate [52]. So, the question remains still debated: Is ICIs administration harmful or beneficial for cancer patients during the COVID-19 pandemic?

The discovery of ICIs literally revolutionized the cancer management. The ICIs are monoclonal antibodies directed against the so-called immune checkpoints which are activated by cancer cells in the attempt to suppress the immune system and its anti-cancer activity [53,54]. ICIs include anti-PD-1, anti-PD-L1 and anti-CTLA-4 antibodies and they represent a revolutionary type of immunotherapy for the treatment of solid tumors, including melanoma, lung cancer, renal carcinoma, urothelial cancers and head and neck cancers, as well as several hematological cancers, alone or in combination between each other or with other cytotoxic chemotherapies [55,56,57]. For their nature, ICIs restore the cellular-mediated immunocompetence. By blocking the checkpoints PD-1/PD-L1 and CTLA4/B7, the cellular mediated anti-tumor activity of the immune system is then successfully restored and the cancer cells can be efficiently eliminated by the immune system [58].

A case study was conducted on two cancer patients with metastatic melanoma, both treated with anti-PD-1 and anti-CTLA4 in combination. They developed immune-related pneumonitis as side effect of the therapy during the pandemic. Importantly, they tested negative to SARS-CoV-2. Both patients, were successfully treated and dismissed from the hospital within a week, and still testing negative to the virus. The authors warranted caution in administering the ICIs to cancer patients (especially if administered as a combination of more than one type), although they did not develop COVID-19 [59].

Another case study conducted on two cancer patients treated with ICIs, who were infected with SARS-CoV-2, developed mild COVID-19 symptoms and they positively recovered from the infection. The finding suggests that treatment with ICIs must not be stopped during the COVID-19 pandemic and the eventuality of a SARS-CoV-2 infection does not obstacle to grant cancer patients with the best standard of care treatment against the infection, which is not associated with a severe symptomatology [60].

Do ICIs compromise cancer patients’ immunity and increase the vulnerability to COVID-19 infection? Or, on the contrary, do ICIs potentiate their immune system? A recent review on this topic has been published on April 15th in Immunotherapy journal, by Kattan et al., highlighting the benefits of administering ICIs in responder cancer patients. Indeed, the patients showed a significant improvement in the cancer prognosis of otherwise untreatable malignancies. Thus, in such cases, ICIs must be regularly administered [61].

As described above, currently, very few data are available on cancer patients affected by COVID-19 and treated with ICIs, so the oncologists are left alone to balance risks and benefits of interruption or continuation of such therapy, on a case-by-case basis.

Two main issues with ICIs are that: (1) a percentage of treated patients do not respond to them and (2) the individual tolerability of cancer patients may differ, as some subjects, in sporadic cases, may develop immune-related adverse events (irAEs) which may vary from mild to life-threatening [62]. The adverse events are rare and extremely heterogeneous, including: Gastrointestinal toxicity, mucosal inflammation, endocrine dysregulation and dermatological abnormalities. Additionally, a 1–5% of irAEs are associated with the development of interstitial pneumonitis, which for some extents, resembles the pneumonitis caused by SARS-CoV-2 [63].

The iaAEs-related pneumonitis risk is very little, but not negligible, for that reason many clinicians prefer to suspend ICI-based treatment in cancer patients during this acute phase of pandemic. Additionally, sporadic data indicate that viral infection or reactivation may be a complication of ICIs administration [64].

A recently opened clinical study (which will be open until the end of the pandemic) is currently collecting data in an open register called TERAVOLT, specific for patients affected by thoracic cancer which are infected with SARS-CoV-2. Importantly, preliminary analyses of the available data suggest that ICI-based therapy does not increase the overall mortality of thoracic cancer patients affected by COVID-19, nor their overall hospitalization rate [30].

**Figure 1:**
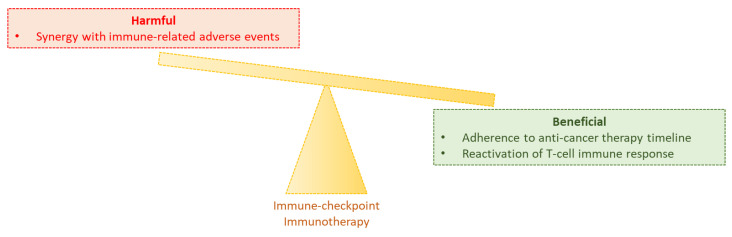
Balance between harmful and beneficial effects of immune-checkpoint immunotherapy in cancer patients during COVID-19 pandemic. If on one side immune checkpoint inhibitors (ICIs) have shown rare immune-related adverse events, in particular interstitial pneumonitis (red box); on the other side cancer patients that do not postpone the immunotherapy, continue to adhere to the program therefore having more chance of positive outcomes. Moreover, they reactivate the immunocompetence, in particular the T-cell mediated immunity, not only towards cancer cells, but also against viral infection (green box).

As said, cytotoxic chemotherapies and radiotherapies may induce myelosuppression, therefore they lower the overall humoral, as well as the cellular mediated immune response [65]. On the contrary, cancer patients treated with ICIs have been demonstrated able to restore their immunocompetence during HIV, hepatitis B or hepatitis C viral infection, thereby suggesting that those individuals might be highly immunocompetent if compared to the other cancer patients subjected to cytotoxic regimens [66]. A summary recapitulating the main advantages and disadvantages of administering ICIs to cancer patients during COVID-19 pandemic, is reported in Figure 1.

## 5. Our Hypothesis: Using ICI-Blockade in Cancer Patients during the Pandemic Does not Harm and Might Be a Game-Changer

The measures adopted for ICIs are currently based on a case-by-case approach. Data regarding the association of ICIs administration and COVID-19 outcomes are currently very few and contrasting. Since the pandemic started, scientific opinions have been diffused, but not enough solid observational data have been generated yet [37]. Importantly, there is no direct evidence of ICI-induced toxicity and increased viral infection risk in cancer patients, prior or during the pandemic. In fact, most cancer care centers agree on continuing ICIs, especially for responder patients with lung cancer [49].

The cytokine release syndrome, which triggers the CS, is a severe complication in patients with COVID-19, as it may induce ARDS [19]. As described above, following a first hyper-inflammatory phase triggered by the CS, SARS-CoV-2 prolonged infection might induce T-cell hyperactivation and finally exhaustion, associated with concurrent patients’ lymphopenia, as well as sepsis [67]. In case of severe SARS-CoV-2 infection, although both CD4+ and CD8+ T-cells in COVID-19 patients start to differentiate, such cells are reduced in abundance and less activated. Consequently, the virus clearance is delayed, because the excessive exhaustion of CD8+ T-cells in severe COVID-19 patients may reduce their cellular-mediated immune response against the virus [68,69]. In detail, impaired peripheral T-cells upregulate both the mucin-3 and PD-1 immunosuppressive markers [67]. As a consequence, severe and critical COVID-19 patients might end up with viral sepsis [70]. Finding a therapy to restore the T-cell functionality in COVID-19 patients might avoid the development of viral sepsis and ARDS complications.

Independent data in mice and in patients, demonstrated that PD-1/PD-L1 blocking antibodies administered to subjects chronically infected with viruses (i.e., lymphocytic choriomeningitis virus in mice or HIV in humans), enhance the viral control and virus-specific CD8+ T-cell responses. This observation demonstrates that the PD-1 inhibitory pathway is particularly important in exhausted T-cells formation. In fact, PD1-blockage restored both CD4+ and CD8+ T-cells abundance and functionality [71,72].

Based on these observations, is it acceptable to continue ICIs to cancer patients during COVID-19 acute pandemic phase? From the above-reported clinical studies, it is not demonstrated that there is any occurring synergy between ICIs-induced pneumonitis and the inflammatory ARDS determined by SARS-CoV-2 that can be lethal. Therefore, we suggest to not suspend the ICI-programmed therapy in cancer patients during the pandemic. Additionally, we advise to consider on a case-by-case basis the eventual association of ICIs with immunosuppressive corticosteroids or chemotherapies. Finally, when possible, we propose to concentrate the therapy sessions, so the cancer patient may limit the entrances in the hospital, which can represent a place at higher risk to contract the SARS-CoV-2 infection.

In general, not only the ICIs-based therapy is pivotal for cancer eradication, especially in patients that are responding to this therapeutic approach. Moreover, we would like to highlight that ICIs might be a possible game-changer for cancer patients during this COVID-19 pandemic. In fact, given the scientific considerations reported above, cancer patients subjected to anti-PD-1 or anti-PD-L1 antibody therapy might restore their T-cell anti-cancer (and possibly anti-viral) immune response. In turn, this can help to fight the SARS-CoV-2 infection.

**Figure 2:**
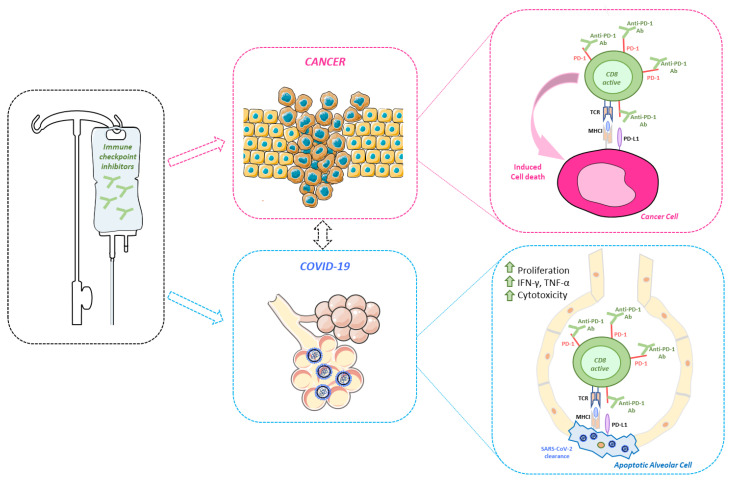
Effect of ICI-immunotherapy on T-cell immunity during cancer (purple boxes) and COVID-19 (light blue boxes). CD8+ T-cells, upon ICI treatment (such as anti-PD-1 antibodies) reactivate the T-cell cytotoxic response again cancer and, potentially, against severe acute respiratory syndrome associated coronavirus-2 (SARS-CoV-2) infection.

In support to our opinion, a registered clinical study is currently ongoing, conducted on metastatic and advanced cancer patients, affected by COVID-19 and which are not eligible to be transferred into an ICU. The study already enrolled 384 patients and, once finished, it will uncover the differential efficacy to eradicate SARS-CoV-2 infection in COVID-19 patients treated either with anti-PD-1 antibody nivolumab in association with standard care protocol, or with standard care alone (NCT04333914, see Table 1). A summary of the potential immune-related benefits of ICI-immunotherapy is schematized in Figure 2.

ICIs might restore individual cellular-mediated immunocompetence and this lesson from cancer may be even transferred to non-cancer COVID-19-affected subjects. In fact, ICIs have already been used beyond cancer to treat, for example, sepsis-induced immunosuppression [73,74]. Moreover, ICIs have been confirmed to be safe when administered to cancer patients vaccinated for influenza virus [75,76]. In line with this concept, three additional independent clinical studies are currently enrolling non-cancer COVID-19 patients to test the efficacy of administering ICIs to reshape the impaired immune system of SARS-CoV-2 infected individuals (i.e., NCT04268537; NCT04356508 and NCT04413838).

Importantly, in order to detect SARS-CoV-2 levels during the progression of COVID-19 and, hence, to evaluate the efficacy of the ICIs-based immunotherapy, the research is currently trying to identify effective and fast viral detection strategies. The RT-PCR based methodologies are the currently recommended standard strategies suggested by the WHO for both SARS-CoV-2 diagnosis and prognosis [77]. In addition, novel high-sensitive molecular methods based on the use of the droplet digital PCR have been proposed for the effective virus detection in COVID-19 patients, even with low viral load [78,79].

## 6. Conclusions

The pandemic is currently rising and the daily reports from the WHO do not leave space to hope for any shortcoming improvement, as 10 million cases and nearly 500,000 deaths of COVID-19 have now been reported globally (Situation report 161, 29 June 2020) [80].

Cancer patients are considered subjects with a higher risk of COVID-19 poorer outcome. The recent clinical data evidenced that the risk to develop severe or critical symptoms of COVID-19 is correlated to factors co-occurring in a cancer patient (e.g., elderly age, sex and presence of many comorbidities), and not to the cancer condition per se.

Moreover, the most recent and bigger reports (in terms of cohort size) evidenced that none of the anti-cancer therapy significantly affected the overall risk of infection or worsen of the COVID-19 symptomatology. Currently, it seems that any anti-cancer regime might be followed without any concern during the pandemic, in contrast with the initial suggestions to interrupt or postpone it, where possible.

Only for a specific therapy, the ICIs the debate is still open and, despite the lack of real evidence, many clinicians decided to interrupt or postpone the ICIs-based immunotherapy in cancer patients, worried of the very remote eventuality that ICI-induced pneumonitis (which represents a very rare side effect) might synergize with ARDS in presence of COVID-19.

Based on the positive effect that ICIs have towards T-cell reactivation against cancer cells, as well as virus-infected cells, ICIs administration may not represent a risk for cancer patients during this pandemic and, in fact, they can be suggested as protective for cancer patients who are infected by the SARS-CoV-2.

## Figures and Tables

**Table 1:** Open clinical studies registered at clinicaltrials.gov on coronavirus disease 2019 (COVID-19) and cancer.

NCT Number	Title	Interventions	Gender	Phases	Enrollment
NCT04392128	Study Evaluating the Efficacy of Hydroxychloroquine and Azithromycine in Patients With COVID-19 and Hematological Malignancies	Hydroxychloroquine; Azithromycin; Placebo	All	Phase 2	114
NCT04341207	Epidemiology of SARS-CoV-2 and Mortality to COVID-19 Disease in French Cancer Patients	Hydroxychloroquine; Azithromycin	All	Phase 2	1000
NCT04447235	Early Treatment With Ivermectin and Losartan for Cancer Patients With COVID-19 Infection	Ivermectin; Losartan; Placebo	All	Phase 2	176
NCT04442048	Immunization With IMM-101 vs. Observation for Prevention of Respiratory and Severe COVID-19 Related Infections in Cancer Patients at Increased Risk of Exposure	IMM-101	All	Phase 3	1500
NCT04333914	Prospective Study in Patients With Advanced or Metastatic Cancer and SARS-CoV-2 Infection	Chloroquine analog (GNS651); Nivolumab; Tocilizumab; Avdoralimab; Monalizumab	All	Phase 2	384
NCT04419623	A Study of TL-895 With Standard Available Treatment Versus Standard Available Treatment for the Treatment of COVID-19 in Patients With Cancer	TL-895; Placebo	All	Phase 1|Phase 2	146
NCT04379518	Rintatolimod and IFN Alpha-2b for the Treatment of Mild or Moderate COVID-19 Infection in Cancer Patients	Recombinant Interferon Alfa-2b; Rintatolimod	All	Phase 1|Phase 2	80
NCT03648372	Evaluation of TAK-981 in Patients With Advanced or Metastatic Solid Tumors or Relapsed/Refractory Hematologic Malignancies and in a Subset With COVID-19	TAK-981	All	Phase 1	80
NCT04370834	Tocilizumab for Patients With Cancer and COVID-19 Disease	Tocilizumab	All	Phase 2	217
NCT04369365	Prophylactic Treatment With Oral Azithromycin Versus Placebo in Cancer Patients Undergoing Antineoplastic Treatment During the COVID-19 Pandemic	Azithromycin; Placebo	All	Phase 2	200
NCT04404361	PRE-VENT Study in Hospitalized Patients With Severe COVID-19 With or Without Cancer	Pacritinib; Placebo	All	Phase 3	358
NCT04381988	A Study of Hydroxychloroquine vs. Placebo to Prevent COVID-19 Infection in Patients Receiving Radiotherapy	Hydroxychloroquine; Placebo; Radiation	All	Phase 2	132
NCT04384588	COVID19-Convalescent Plasma for Treating Patients With Active Symptomatic COVID 19 Infection	Convalescent Plasma from COVID-19 donors	All	Phase 2|Phase 3	100
NCT04426201	InterLeukin-7 to Improve Clinical Outcomes in Lymphopenic Patients With COVID-19	CYT107; Placebo	All	Phase 2	48
NCT04394182	Ultra Low Doses of Therapy With Radiation Applicated to COVID-19	Low-dose radiotherapy; Lopinavir/ritonavir; Hydroxychloroquine; Azithromycin; Piperacillin/tazobactam; Low molecular weight heparin; Corticosteroid injection; Tocilizumab	All	na	15
NCT04439006	Ibrutinib for the Treatment of COVID-19 in Patients Requiring Hospitalization	Ibrutinib	All	Phase 2	72
NCT04446429	Anti-Androgen Treatment for COVID-19	Dutasteride; Ivermectin; Azithromycin	Male	na	254
NCT04445337	Stellate Ganglion Blockade in COVID-19 Positive Patients	Stellate Ganglion Block	All	na	10
NCT04377659	Tocilizumab for Prevention of Respiratory Failure in Patients With Severe COVID-19 Infection	Tocilizumab	All	Phase 2	40
NCT04379492	A Study of Hydroxycholoroquine Compared to Placebo as Treatment for People With COVID-19	Hydroxychloroquine; Placebo	All	Phase 2	120
NCT04402840	Stellate Ganglion Block (SGB) for COVID-19 Acute Respiratory Distress Syndrome (ARDS)	Stellate Ganglion Block	All	na	5
NCT04341480	The Safety of Chemotherapy for Patients With Gynecological Malignancy in High-risk Region of COVID-19	Chemotherapy	Female	na	207
NCT04344002	Lung Cancer Patients and COVID-19	Observational	All	na	200
NCT04382495	The Impact of COVID-19 Pandemic on Cancer Care	Observational	All	na	200
NCT04389996	COVID-19 Pandemic Impact on Patients With Cancer—A Danish Survey	Observational	All	na	5000
NCT04367870	COVID-19 Detection Test in Oncology	Observational	All	na	2500
NCT04354701	COVID-19 and Cancer Consortium Registry (CCC19)	Observational	All	na	1000
NCT04330521	Impact of the Coronavirus (COVID-19) on Patients With Cancer	Observational	All	na	50
NCT04407143	Study of the Immunity of Patients With Lung Cancer and COVID-19 Infection	Observational	All	na	1000
NCT04393974	COVID-19 and Cancer Patients	Observational	All	na	1000
NCT04406844	An Observational Study to Identify the Issues and Challenges in Cancer Patients on Active Treatment During the COVID-19 Pandemic and the Resulting Lockdown	Observational	All	na	150
NCT04384926	Outcomes of Elective Cancer Surgery During the COVID-19 Pandemic Crisis	Observational	All	na	1000
NCT04427280	Cancer: Rapid Diagnostics and Immune Assessment for SARS-CoV-2 (COVID-19)	Observational	All	na	60
NCT04340219	Oncology-patient-reported Anxiety, Mood, and QoL During the COVID-19 Pandemic	Observational	All	na	394
NCT04352556	COVID19-hematological Malignancies: The Italian Hematology Alliance	Observational	All	na	250
NCT04408339	COVID-19 in Cancer Patients: Evaluation of Clinical Course and Impact on Oncological Care Including Biobanking	Observational	All	na	500
NCT04387656	NCI COVID-19 in Cancer Patients, NCCAPS Study	Observational	All	na	2000
NCT04380766	Covid-19 Pandemic and Pancreatic Surgery in Italy	Observational	All	na	700
NCT04432870	Patients’ Preferences About Rescheduling Colonoscopies Delayed Due to COVID-19: Cross Sectional Study	Observational	All	na	200
NCT04447222	Impact of the COVID-19 Pandemic and HRQOL in Cancer Patients and Survivors	Observational	All	na	1242
NCT04385160	Myeloproliferative Neoplasms (MPN) and COVID-19	Observational	All	na	80
NCT04433871	COVID-19 in Pediatric Oncology and Hematology Centers in France	Observational	All	na	300
NCT04351139	Impact of the COVID-19 Pandemic in Gynecological Oncology	Observational	Female	na	400
NCT04445870	Dramatic Changes in Oncology Care Pathway During COVID-19 Pandemic: The French ONCOCARE-COV Study	Observational	All	na	100
NCT04366154	Impact of the COVID-19 on the Management of Oncology and Onco-hematology Patients and on the Psychological Consequences for Patients and Caregivers	Observational	All	na	385
NCT04444401	Registry on NEN Patients and COVID-19	Observational	All	na	50
NCT04374838	Effect of COVID-19 Pandemic on Pediatric Cancer Care	Observational	All	na	20
NCT04363632	Prospective Analysis of Morbi-mortality of Patients With Cancers in Active Phase of Treatment Suspected or Diagnosed of a SARS-CoV-2 Infection	Observational	All	na	150
NCT04416438	COVID-19 Epidemic and Patients With Myeloproliferative Neoplasias	Observational	All	na	50
NCT04366219	Impact of Confinement and Preventive Measures in Period of SARS-COV2 Infection on Patients With Lung Cancer	Observational	All	na	2000
NCT04397575	The GCO-002 CACOVID-19 Cohort: A French Nationwide Multicenter Study of COVID-19 Infected Cancer Patients	Observational	All	na	1000
NCT04345315	Correlative Study on Cancer Patients and Healthcare Professionals Exposed to Infection by SARS-Cov-2	Observational	All	na	500
NCT04406571	Reorganization of the Healthcare System During COVID-19 Pandemic: Impact on Management of Patients With Exocrine Pancreatic Cancer	Observational	All	na	700
NCT04379232	Surgical Activity During the Covid-19 Pandemic: Results for 112 Patients in a French Tertiary Care Center	Observational	All	na	112
NCT04397614	Mobile Health Study and Enhanced Symptom Monitoring in COVID-19 Cancer Patients	Observational	All	na	500
NCT04434261	Oncological Surgery in Times of COVID-19: Effectiveness of Preoperative Screening for Sars-Cov-2	Observational	All	na	1500
NCT04357574	Assessing the System for High-Intensity Evaluation During Radiotherapy During Changes in Response to COVID-19	Observational	All	na	1000
NCT04445402	Pediatrics HOT COVID-19 Database in NY Tristate	Observational	All	na	1500
NCT04401124	Status of Management of Surgery in Beijing During COVID-19	Observational	All	na	500
NCT04389684	Clinical and Psycho-social Impact of COVID-19 Related Confinement on Patients With Digestive Tumors	Observational	All	na	120
NCT04371315	Risk Factors, Clinical Characteristics and Outcomes of Acute Infection With COVID-19 In Children	Observational	All	na	400
NCT04341012	Breath Analysis Based Disease Biomarkers of COVID-19 and Other Diseases	Observational	All	na	120
NCT04385147	Advanced Endoscopy During COVID-19	Observational	All	na	250
NCT04352699	Outcomes of Urological Surgery During Periods of Social COVID-19 Containment: Is it Reasonable to Limit Access to Surgical Care for All?	Observational	All	na	120
NCT04354818	COVID-19 Outcomes Registries in Immunocompromised Individuals Australia (CORIA)	Observational	All	na	1000
NCT04434417	Validation of an Immunochromatographic Assay for IgG/IgM Antibodies to 2019-nCoV	Observational	All	na	1000
NCT04367805	COVID-19 Infection in Patients With Hepatocellular Carcinoma	Observational	All	na	50
NCT04391946	Observatory of Patients With Chronic Lymphocytic Leukemia/Lymphocytic Lymphoma or Waldenstrom Disease Infected With COVID-19	Observational	All	na	50
NCT04386512	Clinical Epidemiology and Characteristics Of COVID-19 Cases Occurred In A Lymphoma Setting In The First Epidemic Phase	Observational	All	na	50
